# WHO PAYS FOR VETERANS’ POSTACUTE SKILLED HOME HEALTH CARE? A COMPARISON AMONG VA, MEDICARE, AND MEDICARE ADVANTAGE

**DOI:** 10.1093/geroni/igae098.2155

**Published:** 2024-12-31

**Authors:** Marguerite Daus, Frank Devone, Emily Corneau, Kate Magid, Katherine Kennedy, Pedro Gozalo, Kali Thomas, Christine Jones

**Affiliations:** Department of Veterans Affairs, Aurora, Colorado, United States; United States Department of Veterans Affairs, Providence, Rhode Island, United States; Providence VA Medical Center, Providence, Rhode Island, United States; Rocky Mountain Regional VA, Aurora, Colorado, United States; VA Providence Healthcare System, Center of Innovation in Long-Term Services and Supports, Providence, Rhode Island, United States; Brown University, Providence, Rhode Island, United States; Johns Hopkins University, Baltimore, Maryland, United States; University of Colorado, Aurora, Colorado, United States

## Abstract

Although costs to the Veteran Health Administration associated with post-acute home health care (HHC) have grown, limited evidence is available about the use and financing of HHC among older Veterans. The objective of this study is to identify Veteran, VA Medical Center (VAMC), and market characteristics that are associated with VHA versus Medicare-funded post-acute skilled HHC for Veterans who are discharged from a VAMC hospital with skilled HHC. We assembled and merged data from several sources for FY 2017-2019 including the VHA’s Residential History File, VHA Corporate Data Warehouse, and Centers for Medicare and Medicaid Services. Our cohort (n=64,347 Veterans, VAMCs=114) included all VHA and Medicare enrolled Veterans, aged 66 years or older, admitted to a VAMC for an acute care hospitalization and who received post-acute home health care within 14 days of discharge. We found that being MA-enrolled increased the probability of the VHA paying for HHC by 28.05% (95% Confidence Interval (CI): 27.12%, 28.96). Other important predictors that increased the probability of VHA-paid HHC include previous VHA-paid HHC episodes, previous VHA inpatient admission in the past 12 months, and an admitting diagnosis of sepsis. The most notable finding was that Veterans who are enrolled in MA have a significantly higher probability of having their post-acute HHC paid for by VHA. We will discuss how differences in HHC cost-sharing between MA and TM for members, prior authorization requirements, and denials of care used by MA plans could explain the lower rates of MA-paid HHC.

